# Development of a novel method to evaluate sialylation of glycoproteins and analysis of gp96 sialylation in Hela, SW1990 and A549 cell lines

**DOI:** 10.1186/s40659-015-0041-8

**Published:** 2015-09-12

**Authors:** Yangui Liang, Qiang Hua, Pengwei Pan, Jie Yang, Qi Zhang

**Affiliations:** State Key Laboratory of Medicinal Chemical Biology, College of Pharmacy, Nankai University, Tianjin, 300071 China; Institute of Information On Traditional Chinese Medicine, China Academy of Chinese Medical Sciences, Beijing, 100700 China; China Center of Industrial Culture Collection, China National Research Institute of Food and Fermentation Industries, Beijing, 100015 China

**Keywords:** Click chemistry, Sialic acid, gp96, Sialyltransferase

## Abstract

**Background:**

Glycoproteins play a critical role in the cellular activities of eukaryotes. Sialic acid is typically the outermost monosaccharide of glycolipids and glycoproteins, and is necessary for normal development.

**Results:**

A strategy based on avidin–biotin affinity was established to enrich sialylated glycoproteins from HeLa cervical carcinoma, SW1990 pancreatic adenocarcinoma, and A549 lung adenocarcinoma cells. Using HPLC–MS/MS, western blot, real-time PCR, and enzyme-linked immunosorbent assay, gp96 was identified in all three cell lines. No significant difference in the protein expression of gp96 was detected at the whole cell level, but the amount of biotinylated gp96 in SW1990 cells was 30–40 % lower than that in A549 and HeLa cells, and the amount of sialylated gp96 in SW1990 cells was 30 % lower than that in A549 and HeLa cells. Immunoblotting results showed that the expression of sialyltransferase proteins in the total cell lysates from HeLa and A549 cells were higher than that in SW1990 cells.

**Conclusions:**

We established a new method for investigating the expression and sialylation of glycoproteins using metabolic labeling, click chemistry, and avidin–biotin affinity. We successfully used this method to purify sialylated glycoproteins from cancer cell lines. Our results showed that the levels of gp96 sialylation varied across different cancer cell lines, and this may be because of differences in sialyltransferase expression.

## Background

Glycosylation is an important post-translational modification of eukaryotic proteins [1]. This modification plays crucial roles in various biological events, such as cell recognition, inter- and intracellular signaling, cell adhesion, and cell–cell interactions [2]. Different levels of glycosylation are often observed in pathological cells compared with normal cells and are regarded as a hallmark of disease states [3]. For example, cancer cells frequently display glycans at different levels or with fundamentally different structures than those observed on normal cells [3]. Sialic acid is typically the outermost monosaccharide unit on the glycan chains of glycolipids and glycoproteins, and is often part of the recognition sites to which pathogens attach [4]. Sialic acid not only provides important intrinsic functions but also is necessary for normal development. The functional significance of changes in the amount of sialic acid in disease-associated states has been reported, and abnormal changes in the amount of sialic acid were observed in prostate, colon, and stomach cancers, among others [4, 5].

Click chemistry is the most practical and reliable set of chemical reactions to connect a diverse range of structures. The Cu(I)-catalyzed azide-alkyne 1,3-dipolar cycloaddition is one of the most common reactions in click chemistry and demonstrates high yield, good efficiency, and high purity [6]. This click reaction also demonstrates excellent selectivity in chemical synthesis [7]. Because the azide and alkyne groups are easily introduced into the structure of molecules, this reaction has been widely used for the fabrication of biological or chemical sensors. For example, the unnatural sugar *N*-a-azidoacetylmannosamine (ManNAz) was metabolized by cultured cells to *N*-a-azidoacetyl sialic acid (SiaNAz) and incorporated into membrane glycoconjugates. Azido-labeled glycoproteins were then reacted with the alkyne probe using click chemistry, and optical imaging indicated a significant azido-labeled *N*-acetylmannosamine-dependent increase on the cell surface [8, 9].

In this study, mannose was modified with an azide group, metabolized by cells, and presented on glycoproteins. Azido-labeled glycoproteins were biotinylated using a Cu(I)-catalyzed [3 + 2] cycloaddition reaction with an alkyne probe. Additionally, a strategy for isolating cell-surface sialylated glycoproteins based on avidin–biotin affinity was established. We used this strategy to identify and evaluate the heatshock protein gp96 as a cell-surface sialylated glycoprotein.

## Results

### Cellular metabolism and biotinylation

**Fig. 1:**
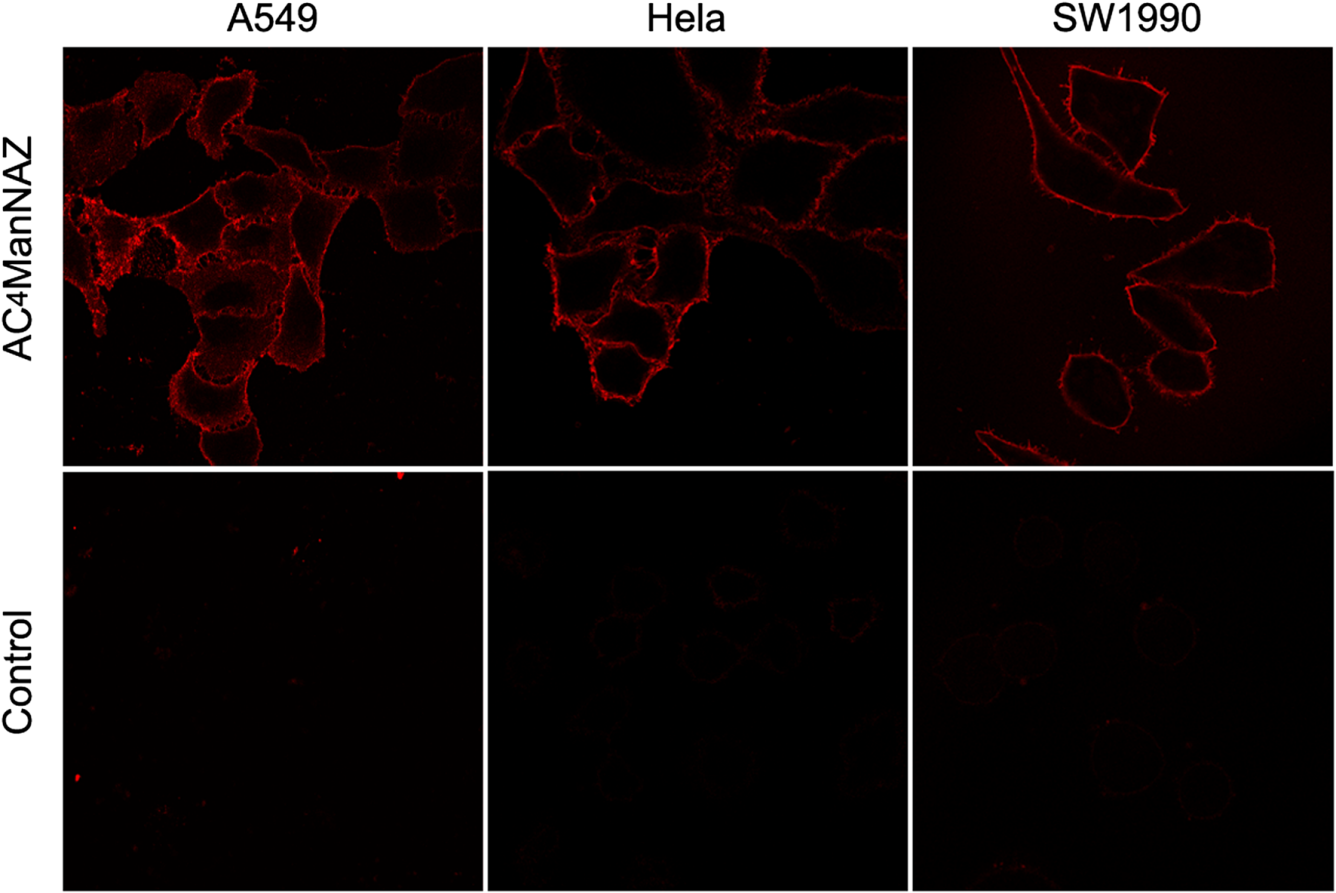
Fluorescent labeling of azido groups on the cell surface

HeLa, SW1990, and A549 cells were cultured for 48 h with 0.2 mM Ac_4_ManNAz to allow the cultured cells to metabolize and incorporate the synthetic Ac_4_ManNAz into membrane glycoproteins. The azides present on the glycoproteins reacted with the biotin-LC-alkyne probe, generating the biotinylated glycoproteins through click chemistry. Cells were stained using avidin-Cy3, and at a 570-nm excitation wavelength, a clear fluorescence signal was observed on the cell surfaces (Fig. 1), which indicated that the cells had metabolized Ac_4_ManNAz. No fluorescence was observed in the control cells. These results suggested that the increased fluorescence resulted from a specific affinity for azido glycans and that the azido groups on the cell surface might undergo a highly selective reaction with the biotin-LC-alkyne probe. Additionally, we did not observe fluorescence within the cells, which indicated that the biotin-LC-alkyne probe cannot penetrate the cell membrane and that the biotinylation only occurred on the cell surface of the surviving cells through click chemistry.

### Isolation of the biotinylated glycoproteins

**Fig. 2:**
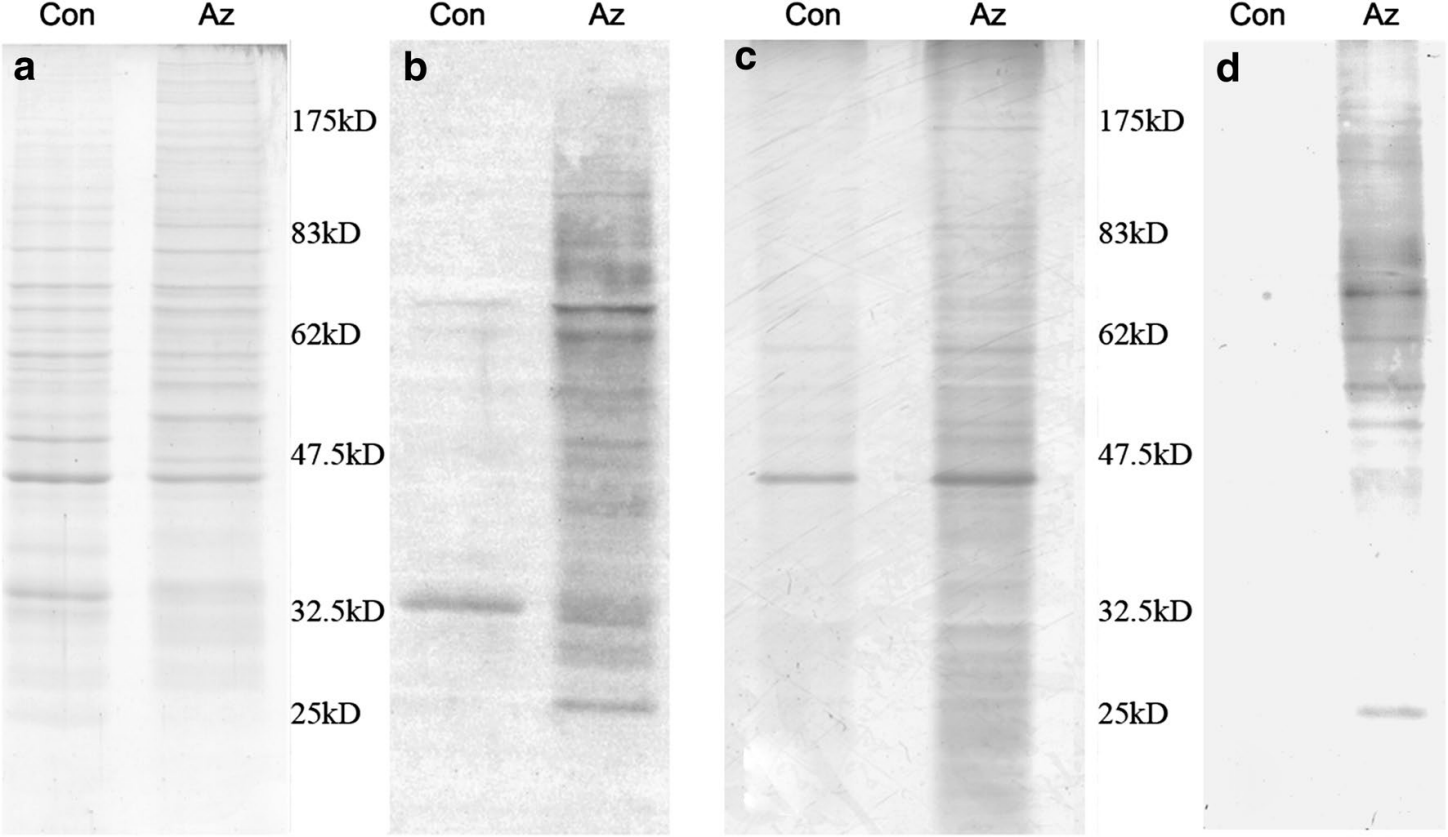
Purification of glycoproteins. **a** Coomassie Brilliant Blue staining of the total cell lysate. **b** Western blot analysis of the total cell lysate using HRP-streptavidin. **c** Coomassie Brilliant Blue staining of the purified proteins. **d** Western blot analysis of the purified proteins using HRP-streptavidin. *Con* control, *Az* Ac_4_ManNAz

After isolation of the glycoproteins present on the cell surface using the biotin- avidin system, the amount of obtained glycoproteins was analyzed using Coomassie Brilliant Blue G-250 staining, and the level of biotinylation was examined. Coomassie staining of the total cell lysates revealed mostly similar protein bands between the labeled and control cells, with only minor differences (Fig. 2a), which suggested that the unnatural sugar did not affect cellular metabolism. In contrast, HRP-streptavidin detected extensive biotinylation of proteins in the labeled cells. Figure 2b demonstrates an equal molecular weight distribution. This result suggested that the azido groups were effectively metabolized and specifically biotinylated. SDS-PAGE analysis of the purified proteins that were isolated using streptavidin magnetic microspheres (SMMs) indicated many specific protein bands in the labeled cells compared with the control cells (Fig. 2c). Additionally, the azido-labeled proteins could be specifically biotinylated in contrast to the control cells (Fig. 2d). Collectively, these results indicated that the metabolized synthetic sugar was incorporated into glycoproteins and it could be specifically biotinylated and detected. Furthermore, the corresponding glycoproteins could be isolated using SMMs for subsequent analysis.

### The expression of gp96 in different cancer cell lines

**Fig. 3:**
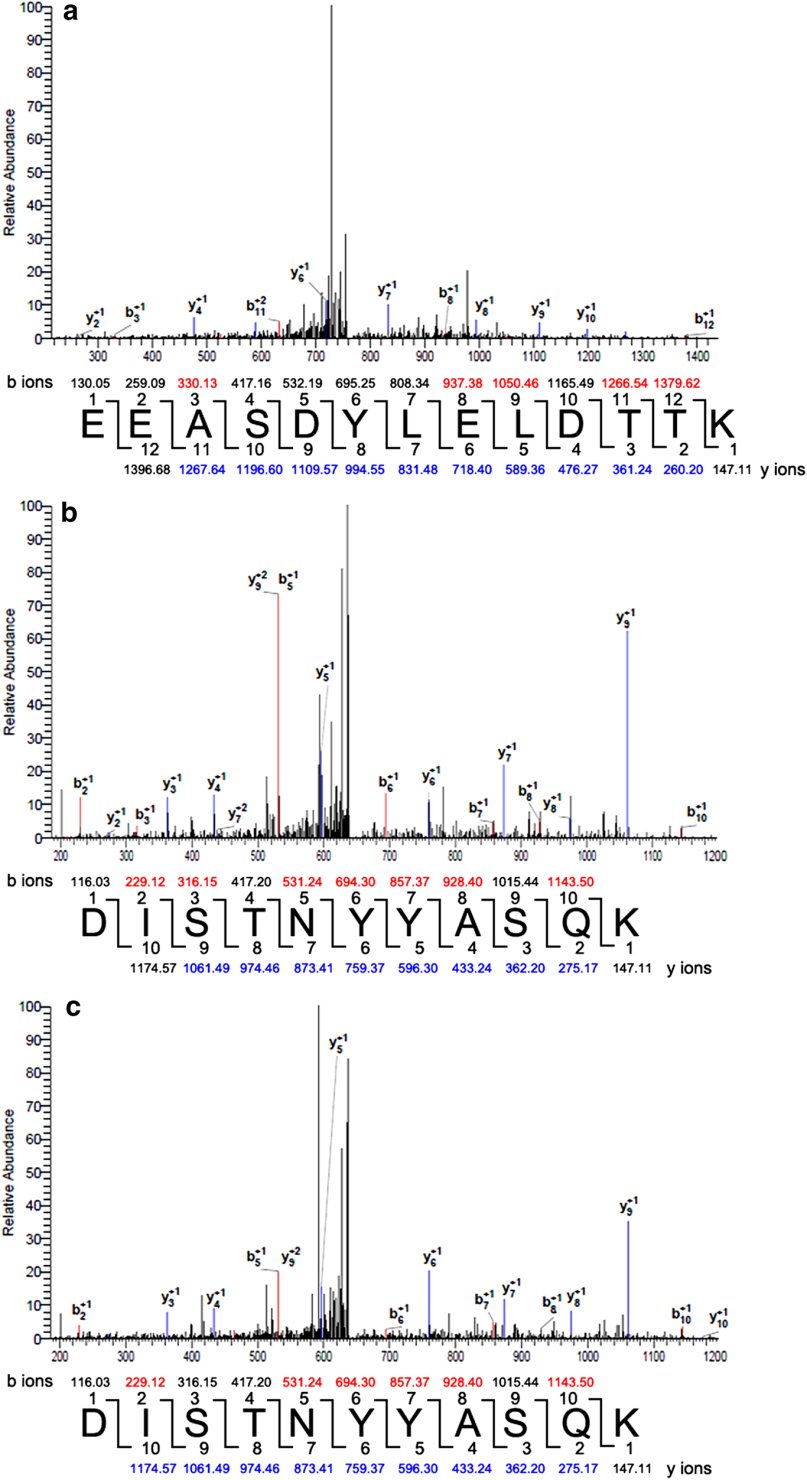
Identification of gp96 using HPLC–MS/MS. MS/MS spectra indicating peptide sequences of gp96 that were detected in **a** HeLa, **b** A549, and **c** SW1990 cells

**Fig. 4:**
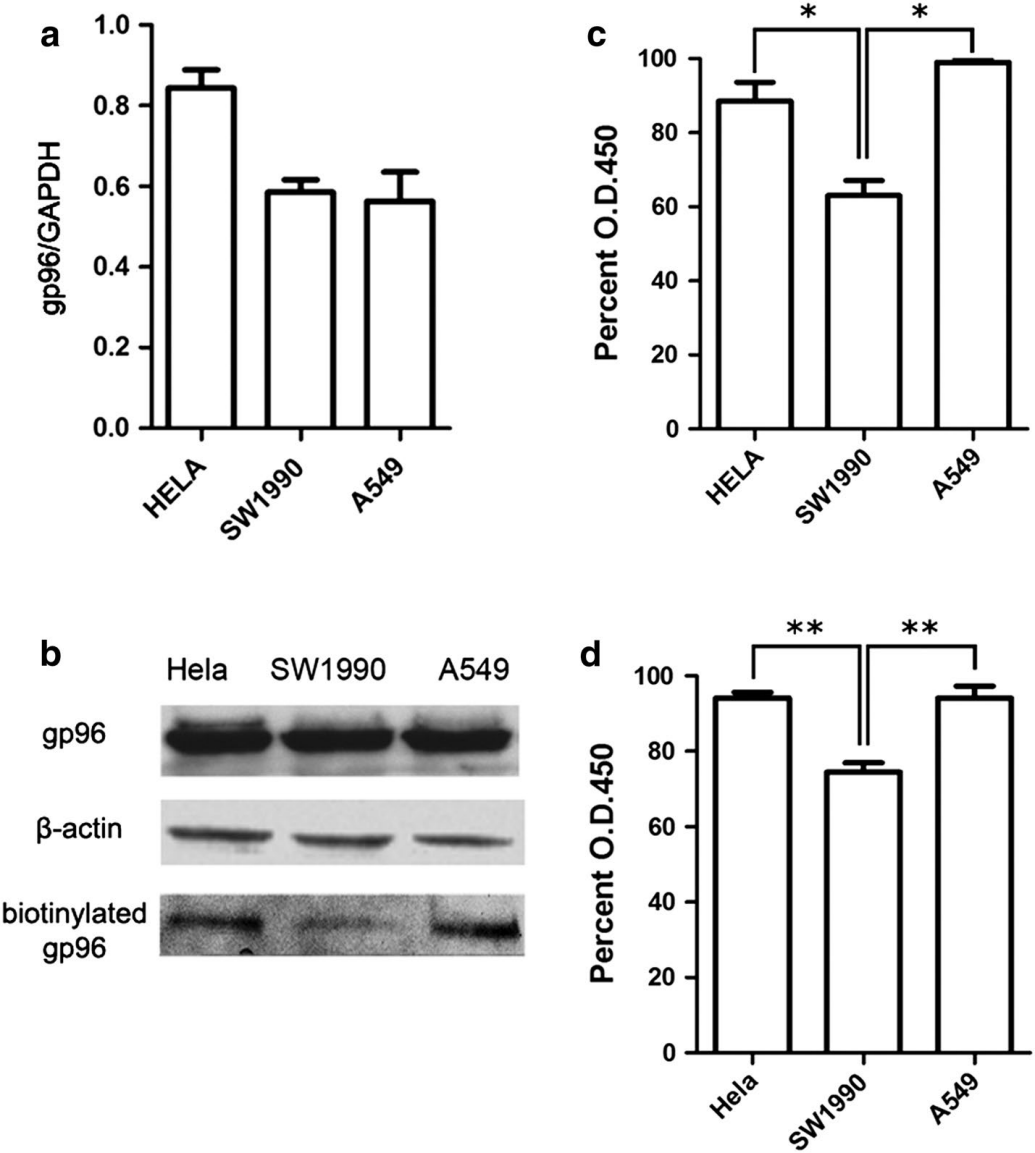
Expression, biotinylation, and sialylation of gp96 in different cancer cell lines. **a** The expression of gp96 mRNA in different cancer cell lines was determined using real-time PCR and normalized to GADPH expression. **b** The expression of gp96 protein and amount of biotinylated gp96 obtained after purification from different cancer cell lines were determined using western blot analysis. β-actin served as an internal control. **c** The variation in the degree of biotinylation of gp96 was determined using ELISA. **d** The variation in the degree of sialylation of gp96 was determined using ELISA

The glycoproteins were isolated from labeled HeLa, A549, and SW1990 cells and analyzed using HPLC–MS/MS. The results identified the gp96 heat shock protein in each cell line (Fig. 3). Real-time PCR and western blot analysis confirmed these results. Real-time PCR revealed that the expression of gp96 mRNA was 30 % higher in HeLa cells than that in A549 and SW1990 cells, using GAPDH as an internal control (Fig. 4a), However, western blot analysis indicated no significant difference in gp96 protein expression in all three cell lines (Fig. 4b). One possible explanation may be that efficiency or regulation of translation may have affected the level of the expressed protein. Despite the discrepancy in the two results, the western blot results provide the actual levels of expressed cellular protein. Simultaneously, the variations were observed in the western blot for biotinylated gp96 (Fig. 4b).

### Variations in sialylation of gp96 in different cancer cell lines

The amount of gp96 in the purified glycoprotein sample was detected using western blot analysis. As shown in Fig. 4b, the amount of purified gp96 protein by SMMs was clearly lower in SW1990 cells than in HeLa and A549 cells. Additionally, enzyme-linked immunosorbent assay (ELISA) was used to measure the level of gp96 biotinylation in the different cell lines. We observed that the level of biotinylated gp96 in SW1990 cells was only 71 and 64 % of that in HeLa and A549 cells, respectively (*P* < 0.05) (Fig. 4c). The lower level of biotinylated gp96 in SW1990 cells resulted in a lower amount of gp96 in the purified glycoprotein sample. Differences in the morphology of adherent cells and growth rates of the different cell lines precluded the evaluation of gp96 expression on the cell surface. Therefore, ELISA was used to determine the sialylation level of gp96 in the different cancer cell lines using HRP-WGA. As shown in Fig. 4d, the level of sialylated gp96 in SW1990 was 79 % of that in A549 and HeLa cells (*P* < 0.01), which suggested that the sialylation level of gp96 differs in different cancer cell lines and indicated that the sialylation level of gp96 or perhaps other glycoproteins may play an important role in tumorigenesis and development.

### Expression of sialyltransferase in different cancer cell lines

**Fig. 5:**
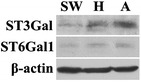
Expression analysis of sialyltransferase. Western blot analysis of sialyltransferase expression in different cancer cell lines was performed using anti-ST3Gal antibody and anti-ST6Gal1 antibody. *SW* SW1990 cells, *H* Hela cells, *A* A549 cells

To investigate the observed differences in sialylation among the cell lines, we performed western blot analysis on the same concentration of total proteins from the three cell lines to analyze the expression of glycosyltransferases related to sialylation, including alpha-2,3-sialyltransferase (ST3Gal) and alpha-2,6-sialystransferase 1 (ST6Gal1). As shown in Fig. 5, the expression of ST3Gal and ST6Gal1 in the total cell lysates from Hela and A549 cells were obviously higher than in SW1990 cells, which reflected the similar trend in sialylation level of gp96 in the three cell lines. These results indicated that the difference of sialyltransferase may be one important reason resulting in the variations in sialylation of gp96 in different cell lines.

### Levels of sialylated gp96 in two breast cancer cell lines

**Fig. 6:**
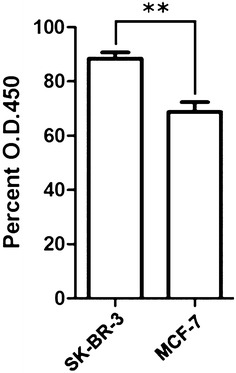
Variations in sialylation of gp96 between MCF-7 and SK-BR-3 cells

To investigate specifically reference differences in metastasis capability between the cell lines, the sialylation of gp96 was analyzed in MCF-7 and SK-BR-3 cell lines, both from breast cancer tissues. As shown in Fig. 6, ELISA results revealed that levels of sialylated gp96 in SK-BR-3 cells were higher than in MCF-7 cells, which was consistent with metastasis capability. These results indicated that the difference of sialylated levels may affect the metastasis potential of tumor cells.

## Discussion

Glycoproteins play very important roles in cellular activities. Currently, several methods for isolating glycoproteins are widely used, including lectin affinity chromatography, and hydrazide chemistry, among others. Lectins have been chosen for the enrichment and visualization of glycoproteins for decades, and lectin-based capture of glycopeptides and glycoproteins has become a desirable method for glycoproteomic analysis [10]. The hydrazide capture method involves the initial oxidation of sugar residues in the glycosylated peptides using periodate before reaction with a hydrazide resin [11]. Click chemistry offers a novel method to label glycoproteins. Using specifically modified sugars, click chemistry enables a more specific isolation of labeled glycoproteins than lectin-based capture methods. In our study, mannose was modified with an azide group, metabolized by cells and incorporated instead of the natural sugars into glycoproteins that were presented on the cell surface. The resulting sialylated glycoproteins present on the cell surface were subsequently isolated and purified. Our strategy offers a new method for investigating the expression and sialylation of glycoproteins and a new tool for glycoproteomics.

Our strategy for isolating cell-surface sialylated glycoproteins was applied to three different cancer cell lines: A549 lung adenocarcinoma, HeLa cervical carcinoma, and SW1990 pancreatic adenocarcinoma cells. Glycoprotein fractions were then analyzed using HPLC–MS/MS and the results identified the gp96 protein in the glycoprotein fraction of all three cell lines. The endoplasmic reticulum-resident chaperone protein gp96 belongs to a class of conserved molecular chaperones known as heat shock proteins and is a member of the HSP90 family. Gp96 is nonpolymorphic and abundant, and it plays important roles in the secretory pathway in maintaining protein homeostasis [12]. Enzyme-linked immunosorbent assay (ELISA) measured the levels of biotinylation and sialylation of gp96 in the different cell lines, and revealed variations in the extent of sialylation of gp96 in different cancer cell lines. These results may suggest different immunological and pathological properties of gp96 in pancreatic adenocarcinoma cells. Although a clear difference was observed, further study is necessary to confirm these results and elucidate the mechanism for the difference in sialylation; for example, several types of glycosyltransferases are related to sialylation, and the expression of glycosyltransferases in cells and their corresponding activities could affect the degree of sialylation [13–17].

Gp96 is found on the surfaces of malignant cells and possesses sialylated *N*-glycans [18]. With the negative charge on the surface of the glycan imparted by the presence of sialic acid residues, sialic acid is a significant terminal glycan modification that impacts the potential to inhibit intermolecular and intercellular interactions. Other reports showed that aberrant sialylation could promote the invasion and adhesion of cancer cells; higher sialic acid content was correlated with cell attachment and migration, thus contributing to the migratory and invasive phenotype of the cells. A previous study reported that the amount of sialylated glycans on gp96 was significantly lower in regressing cells than that in progressively growing cells. Moreover, the aberrations in the sialic acid content of gp96 can affect its essential protein–protein interaction with antigen presenting cell surface receptors, thus affecting the immunogenicity of gp96 [19]. In our study, we found that the sialylation level of gp96 varied across different cancer cell lines, which might affect the immunological properties of gp96.

In mammals, sialic acids are found at the non-reducing terminal residue of oligosaccharides as α-2,3- or α-2,6-linked to a α-d-galactopyranosyl (Gal), or α-2,6-linked to a α-d-*N*-acetylgalactosaminyl (GalNAc) or a α-d-*N*-acetylglucosaminyl (GlcNAc), the patterns commonly found in glycoproteins [20]. Sialic acids are also found α-2,8-linked to other sialic acids of gangliosides and in polysialic acid. The linear homopolymer has also been observed on several glycoproteins including the neural cell adhesion molecule [21].

Because sialylation is related to glycosyltransferases, studies of glycosyltransferases, including the quantification of the glycosyltransferases present in cells and determination of their corresponding activities, are important. A large amount of sialyltransferase was found in animal cells, which presented in different tissues or cells and performed different functions. Because α-2,8-linked sialic acid homopolymers were found in gangliosides, polysialic acid, and the neural cell adhesion molecule [22], we only investigated α-2,3- or α-2,6-linked sialic acid homopolymers.

To further investigate the differences in sialylation of gp96, the expression of glycosyltransferases related to sialylation, including ST3Gal and ST6Gal1, were examined by western blot. The expression levels of ST3Gal and ST6Gal1 in A549 and Hela cells were higher than SW1990 cells, which was in agreement with sialylation levels and indicated that the higher sialylation may be due to the differences in expression levels of sialyltransferase. Additional studies are currently underway, and the specific influence of immunity and tumor cell migration are under investigation.

Furthermore, variations in sialylation of gp96 in MCF-7 and SK-BR-3 cell lines were analyzed. The result showed that the difference of sialylated levels was agree with the metastasis capability.

We speculate that the difference in the sialylation level of gp96 from different cancer cell lines might affect cellular behaviors, including proliferation, metastasis, differentiation, and other processes, and necessitates further investigation.

## Conclusions

In our study, we established a new method for investigating the expression and sialylation of glycoproteins using metabolic labeling, click chemistry, and avidin–biotin affinity. We successfully used this method to isolate and purify the sialylated glycoproteins present on the cell surface of three different cancer cell lines. Our results showed that the sialylation level of gp96 varied across different cancer cell lines, and this may be due to differences in sialyltransferase expression in different cell lines. Furthermore, the level of sialylation may reflect tumor metastasis ability.

## Methods

### Compound synthesis

Ac_4_ManNAz was synthesized following the protocol described by Srinivasa-Gopalan Sampathkumar [23]. In this study, biotin-LC-alkyne analogs were synthesized using the modified commercialized reagent sulfo-NHS-LC-biotin (Pierce Biotechnology, Rockford, IL, USA), which underwent reaction with propargylamine in borate buffer (0.2 M, pH 8) to modify one end of the long chain with an alkyne. HeLa, SW1990, A549 cells MCF-7 and SK-BR-3 cells were obtained from ATCC (USA).

### Biotinylation of azido-labeled glycoproteins

HeLa, SW1990, and A549 cells were cultured for 48 h, at 37 °C and 5 % CO_2_ in DMEM medium (10 % FBS, 100 units·mL^−1^ penicillin, and 100 ng mL^−1^ streptomycin) supplemented with 0.2 mM Ac_4_ManNAz. As a control, an identical volume of PBS was added to the culture medium. Cells were washed three times with ice-cold 0.1 M PBS (phosphate buffer and 0.15 M NaCl, pH 7.4) and then treated with 1 mL of reaction solution (0.2 mM biotin-LC-alkyne, 0.2 mM Tris-triazoleamine catalyst, 1.0 mM CuSO_4_, and 2.0 mM sodium ascorbate in ice-cold PBS). After incubation for 1 h at 4 °C, the cells were fixed in 4 % paraformaldehyde for 30 min and then washed three times with PBS. Cells were stained using 1 mL of a 1:1000 dilution of avidin-Cy3 (Boster, Wuhan, China) at room temperature for 30 min. After washing samples with PBS carefully, fluorescence images were obtained using laser scanning confocal microscopy.

### Purification of biotinylated glycoproteins

Cells were cultured in Ac_4_ManNAz for 48 h as previously described and treated with biotin-LC-alkyne at 4 °C. After incubation for 1 h, the reaction solution was removed, and the cells were carefully washed with ice-cold PBS. The cells were scraped into ice-cold hypotonic buffer (10 mM HEPES, pH 7.5, 1.5 mM MgCl_2_, 10 mM KCl, 1× protease inhibitor cocktail, 1 mM NaF, and 1 mM Na_3_VO_4_), incubated on ice for 15 min, and then lysed by ultrasonication. After centrifugation at 1000*g* and 4 °C for 10 min, the supernatant was collected. Next, KCl was added to a concentration of 150 mM. Protein concentrations were quantified and diluted to 1 mg mL^−1^. SMMs were added at a volume that was 1/10 of the volume of the supernatant, and the samples were incubated at 4 °C for 1 h. Finally, SMMs were carefully and separately washed with ice-cold PBS and 1 M KCl, suspended in 1× SDS-PAGE loading buffer, and boiled for 10 min, resulting in the purified glycoprotein samples.

### Western blot

The purified glycoproteins were separated using SDS-PAGE and transferred onto a PVDF membrane. Membranes were blocked in 4 % nonfat dry milk for 1 h at room temperature (RT). A 1:1000 dilution of HRP-labeled streptavidin (Boster, Wuhan, China) was added, and the membrane was incubated at RT for 1 h. Biotinylated glycoproteins were detected using electrochemiluminescence (ECL). To detect gp96 protein expression, the membrane was incubated with a 1:1,000 dilution of an anti-gp96 antibody (Santa Cruz, Dallas, USA) overnight at 4 °C followed by incubation with an HRP-labeled secondary antibody for 1 h. Sialyltransferase was analyzed using 1:1,000 dilution of anti-ST3Gal (Santa Cruz) and 1:1000 dilution of anti-ST6Gal1 (Immunoway, Newark, USA) as primary antibodies. Protein expressions were detected using ECL. β-actin served as an internal control and a 1:1000 dilution of anti-β-actin antibody was used.

### HPLC–MS/MS

HPLC–MS/MS and the analysis were performed by Shanghai Applied Protein Technology Co., Ltd.

### Real-time PCR

The mRNA of the different cell lines was isolated using a PolyATtract System 1000 (Promega, Madison, USA) and reverse transcribed to cDNA using a PrimeScript RT reagent kit (Takara, Dalian, China). Real-time PCR was performed using an SYBR Premix EX *Taq* II kit (Takara). The primers used in this study were as follows: gp96 forward primer, 5′-TGT TTC CCG CGA GAC TCT TC-3′; gp96 reverse primer, 5′-CAC ACC AGC ACT GCT GTG CAC CAC AGT GG-3′; GAPDH forward primer, 5′-GTG AAG GTC GGA GTC AAC G-3′; and GAPDH reverse primer, 5′-TGA GGT CAA TGA AGG GGT C-3′.

### ELISA

A 96-well plate was coated with a 1:1000 dilution of an anti-gp96 antibody at 37 °C for 4 h and subsequently blocked with 1 % normal goat serum at 4 °C overnight. After careful washing with PBS, purified glycoproteins (0.5 mg mL^−1^) were added and incubated at 37 °C for 1 h. After washing with PBS, a 1:1000 dilution of HRP-streptavidin or a 1:1000 dilution of HRP-WGA (Sigma, St. Louis, MO, USA) was added and incubated at RT for 1 h. Finally, the signal was visualized using TMB as a chromogen. To detect sialyation of gp96 in MCF-7 and SK-BR-3 cells, we performed ELISA using HRP-WGA.
